# Measures, Uncertainties, and Significance Test in Operational ROC Analysis

**DOI:** 10.6028/jres.116.003

**Published:** 2011-02-01

**Authors:** Jin Chu Wu, Alvin F. Martin, Raghu N. Kacker

**Affiliations:** Information Access Division, National Institute of Standards and Technology, Gaithersburg, MD 20899; Applied and Computational Mathematics Division, National Institute of Standards and Technology, Gaithersburg, MD 20899

**Keywords:** biometrics, bootstrap, confidence interval, fingerprint, ROC analysis, significance test, standard error, uncertainty

## Abstract

In receiver operating characteristic (ROC) analysis, the sampling variability can result in uncertainties of performance measures. Thus, while evaluating and comparing the performances of algorithms, the measurement uncertainties must be taken into account. The key issue is how to calculate the uncertainties of performance measures in ROC analysis. Our ultimate goal is to perform the significance test in evaluation and comparison using the standard errors computed. From the operational perspective, based on fingerprint-image matching algorithms on large datasets, the measures and their uncertainties are investigated in the three scenarios: 1) the true accept rate (*TAR*) of genuine scores at a specified false accept rate (*FAR*) of impostor scores, 2) the *TAR* and *FAR* at a given threshold, and 3) the equal error rate. The uncertainties of measures are calculated using the nonparametric two-sample bootstrap based on our extensive studies of bootstrap variability on large datasets. The significance test is carried out to determine whether the difference between the performance of one algorithm and a hypothesized value, or the difference between the performances of two algorithms where the correlation is taken into account is statistically significant. Examples are provided.

## 1. Introduction

The receiver operating characteristic (ROC) analysis is an important statistical technique in many areas, such as biometrics, medical decision making, etc. [1, 2]. Sampling variability can result in uncertainties of performance measures in ROC analysis. In other words, if a sample set is changed under the same conditions, the measures will vary accordingly. Thus, when evaluating and comparing the performance of algorithms, the measurement uncertainties must be taken into account. The key issue is how to calculate the uncertainties of measures in ROC analysis. Our ultimate goal is to perform the significance test in evaluation and comparison using the standard errors computed. The methods explored in this article can have wide application in different areas, such as biometrics, speaker recognition evaluation, and so on. But in this article, the fingerprint-image matching algorithms were taken as examples for illustration.

**Fig. 1:**
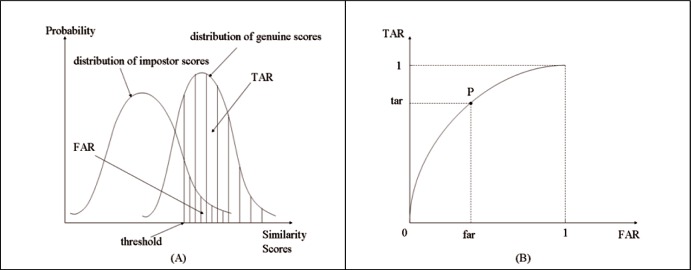
(A): A schematic diagram of two distributions of continuous genuine scores and impostor scores, showing three related variables: *TAR*, *FAR*, and threshold. (B): A schematic drawing of an ROC curve constructed by moving the threshold from the highest similarity score down to the lowest one.

Generally speaking, for instance in biometrics, genuine scores are created by comparing two different images of the same subject, and impostor scores are generated by matching two images of two different subjects. Both scores may be referred to as similarity scores. Notice that similarity scores must be generated by matching the same finger, e.g., right-index finger, or left-index finger, etc., or scores might be created by two-finger fusion [3, 4]. These two sets of similarity scores constitute two distributions, respectively, as schematically depicted in Fig. 1 (A) for continuous similarity scores.

The cumulative probabilities of genuine and impostor scores from the highest similarity score to a specified similarity score (i.e., threshold) are defined as the true accept rate (*TAR*) and the false accept rate (*FAR*), respectively. Thus, in the *FAR*-and-*TAR* coordinate system, as the threshold moves from the highest similarity score down to the lowest similarity score, an ROC curve is constructed as drawn in Fig. 1 (B).

Any point P on an ROC curve has two coordinates *FAR* and *TAR* and is associated with a threshold through two distributions of genuine scores and impostor scores. The three variables, *FAR*, *TAR*, and threshold, are related to each other, as illustrated in Fig. 1 (A) and (B). Any one of them can determine the other two. In practice, it is never required that *TAR* be specified in the first place. Thus, the metrics in the three scenarios are of interest: 1) *TAR* at a specified *FAR*, 2) *TAR* and *FAR* at a given threshold, and 3) the equal error rate (*EER*) where 1—*TAR* (i.e., the probability of type I error) and *FAR* (i.e., the probability of type II error) are equal [5, 6]. The methods of computing the measures in these three scenarios will be provided. The use of these performance metrics to evaluate matching algorithms is referred to as operational ROC analysis.

An ROC curve can also be measured by the area under the ROC curve (*AURC*) [3, and references therein]. If the trapezoidal rule is employed, this area is equivalent to the Mann-Whitney statistic formed by genuine and impostor scores. Hence, the variance of the Mann-Whitney statistic can be utilized as the variance of *AURC*. Since the Mann-Whitney statistic is asymptotically normally distributed, the *Z* statistic can be used to test the significance of the difference between two ROC curves.

**Fig. 2:**
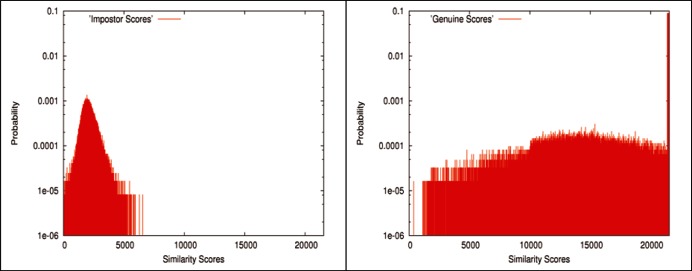
The discrete probability distributions of the genuine scores (right) and the impostor scores (left) generated by a matching algorithm. The integer scores used by this algorithm run from 0 to 21 383. A stand-alone peak at the highest score occupies 8.95 % of the whole population of genuine scores.

As an example, in Fig. 2 are depicted the discrete probability distributions of genuine and impostor scores generated by a matching algorithm. The integer scores used by this algorithm run from 0 to 21 383. This algorithm creates a little over 60 000 genuine scores and a little over 120 000 impostor scores. Hence, the probability is depicted in logarithmic scale. The genuine scores have a stand-alone peak at the highest score occupying 8.95 % of the whole population, and the probability distribution of the impostor scores is a normal-like distribution skewed towards higher scores. Additional such examples can be found in Refs. [3, 4].

This example shows that the distributions of genuine scores and impostor scores usually do not have well defined parametric forms and the shapes of these two distributions for a given algorithm may be considerably different. Also the distributions may vary substantially from algorithm to algorithm, which differentiates algorithms in terms of matching accuracy [3]. An ROC curve is characterized by the relative relationship between these two distributions [3, 4]. This suggests that the nonparametric statistical analysis may be appropriate for evaluating fingerprint-image matching algorithms applied to large-scale datasets. Hence, the empirical distribution is used for each of the observed similarity scores.

Furthermore, the two distribution functions of genuine scores and impostor scores are indeed interrelated by the algorithm that generates them. In other words, the performance of a matching algorithm is affected not only by genuine matching but also by impostor matching. All statistics of interest in ROC analysis are influenced by the combined impact of these two sets of samples. While analyzing data, the probability distribution functions of similarity scores are all discrete after converting scores to integers if they are not so already, and thus the ROC curve is not a smooth curve [3]. It is assumed that an ROC curve discussed in this article is formed using the trapezoidal rule.

The uncertainties of measures in all three scenarios in terms of standard errors (SE) and 95 % confidence intervals (CI) are computed using the nonparametric two-sample bootstrap [7–10] based on our extensive investigation of bootstrap variability on large fingerprint datasets. The two sets of samples are referred to as a set of genuine scores and a set of impostor scores.

The one-sample bootstrap method assumes that an independent and identically distributed (i.i.d.) random sample of size *n* is drawn from a population with its own probability distribution. The i.i.d. assumption is also applied to the two-sample bootstrap method. Our large government databases used for developing similarity scores were randomly collected from real practice rather than obtained from multiple biometric acquisitions of a number of subjects, and thus had no dependencies. The SEs of *AURC* on our databases computed using the nonparametric two-sample bootstrap with the i.i.d. assumption matched very well the analytical results using the Mann-Whitney statistic [11]. Moreover, an example was made, in which the similarity scores were created using the random generator of normal distribution “rnorm” in R [12]. Certainly, there is no dependency among these scores at all. The result shown in the example behaved in exactly the same way as the results derived from our databases. As a result, in our work, the random sample is treated as i.i.d..

With the i.i.d. assumption, the units of a nonparametric two-sample bootstrap are scores in the sample. As pointed out in Ref. [5], if the database had dependencies due to multiple biometric acquisitions, then the i.i.d. assumption could not be made. Then, the sample may need to be grouped into subsets according to dependencies, and the objects of nonparametric two-sample bootstrap would be subsets of the sample in order to preserve the dependencies [10, 13, 14]. However, everything else in the bootstrap method remains intact. Of course, how the sample is grouped into subsets will have impact on the bootstrap results.

In this article, the total number of genuine scores is a little over 60 000 and the total number of impostor scores is a little over 120 000. As demonstrated in our previous studies of sample size in fingerprint applications, if the numbers of similarity scores get larger than these, the measurement accuracy will improve little [15]. The research was carried out by applying the Chebyshev’s inequality to the two metrics: the *AURC* and the *TAR* at a specified *FAR*. With this number of impostor scores, if the *FAR* is set to be 0.001 in Scenario 1, then the number of false-accept instances would be about 120, which is reasonably large in operational practice [4, 15].

Regarding the significance test in ROC analysis, the first category is the one-algorithm significance test related to evaluation, which is to determine whether the difference between the performance of a single algorithm and a hypothesized value is real or by chance. The second category is the two-algorithm significance test related to comparison, which is to investigate whether the difference between the performances of two algorithms is statistically significant. The second category can be extended, for example, if the performances of two different algorithms on the same dataset are replaced by the performances of a single algorithm on two different datasets.

While performing the comparison between two matching algorithms, the metric *TAR* at a given *FAR* and the metric *EER* are typically employed. It is impossible to reach conclusion using *TAR* (the larger the better) and *FAR* (the smaller the better) at a specified threshold simultaneously, if both *TAR* and *FAR* of an algorithm were larger (or smaller) than those of another algorithm.

Such comparison issues can be dealt with intuitively to some extent using 95 % CIs. But it is hard to reach any conclusion while the 95 % CIs overlap for two-algorithm significance test. Nonetheless, such an approach cannot provide any quantitative information, such as how much the *p*-value is, i.e., what the statistical significance of the difference is. Thus, the issue of determining whether the difference is real or by chance must be dealt with using the statistical hypothesis testing.

It is hard to prove the normality of the distribution of the statistics of interest in our applications using the central limit theorem. For instance, for the metric *TAR* at a given *FAR*, the genuine scores at the threshold determined by the given *FAR* may have ties, and those genuine scores at the threshold must be divided proportionally according to the trapezoidal rule in order to compute the *TAR* [5, 6].

However, the relationship between the two types of 95 % CIs for the statistics TAR at a given *FAR* and EER was examined in all cases encountered in Ref. [5, 6]. One type of 95 % CI was computed using the definition of quantile; another type of 95 % CI was calculated if the distribution of bootstrap replications of the statistic was assumed to be normal. It was found that these two types of 95 % CIs were matched up to the third to fourth decimal place. The higher the accuracy of algorithm is, the more decimal places are matched. Moreover, the Shapiro-Wilk normality test [12] was conducted on the bootstrap replications of the statistics of interest, and it was observed that the majority of *p*-values were greater than 5 %, especially for relatively high-accuracy algorithms.

All these suggest that the statistics of interest in our applications are normally distributed regardless of the distributions of genuine and impostor scores. Thus, the *Z*-test will be used to determine the statistical significance of the difference in two categories, as it was done for *AURC* [3, and references therein]. In the case that the alternative hypothesis is accepted, the sign of the difference is employed to determine which is better than the other. In ROC analysis, we do not know beforehand the correlated pairs of metrics, such as *TAR* for a given *FAR*, or *EER*, on which the hypothesis testing is conducted. Thus, the paired t-test cannot serve our purpose.

Bootstrap methods have been applied widely for estimating measurement uncertainties, and so is the use of ROC analysis. Numerous references can be found [14, 16–23, and references therein]. However, employing the methods of nonparametric two-sample bootstrap in ROC analysis can be found in medical applications and the *Z*-test was conducted on *AURC* [17–23].

In medical applications, sizes of data are small. In our applications, such as biometrics and speaker recognition, etc., the sizes of datasets are much larger. For instance, in the fingerprint applications, tens and hundreds of thousands of similarity scores are used. Moreover, in comparison with other applications of bootstrap methods, our statistics of interest are probabilities, such as *TAR*, *FAR*, *EER*, etc., rather than a simple arithmetic mean [5, 6, 10] and our data samples of similarity scores have no parametric model to fit as stated above [3, 10]. Hence, the bootstrap variability was re-studied to determine the appropriate number of bootstrap replications in our applications, in order to reduce the bootstrap variance and ensure the accuracy of the computation [5].

Further, in medical applications, the metric that is used most is *AURC* due to small size of data. From the operational perspective, the measures and accuracies of the statistics of interest, such as *TAR*, *FAR*, *EER*, etc., in all three scenarios were computed using the nonparametric two-sample bootstrap [6]. The *Z*-test was applied on *TAR* and *EER*. An algorithm for computing the correlation coefficient involved in the *Z*-test in our applications is provided. The way of computing correlation coefficient in this paper is completely different from the way in Ref. [17], which is based on a table provided by other researchers. Our methods can also be applied to *AURC* as well as a cost function defined, for instance, as a weighted sum of the probabilities of type I error and type II error in the speaker recognition evaluation [24].

The formulations of discrete probability distributions of genuine and impostor scores, as well as ROC curve are presented in Sec. 2. The methods for calculating the measures of statistics of interest in three scenarios are shown in Sec. 3. The nonparametric two-sample bootstrap algorithms of computing their uncertainties are provided in Sec. 4. The empirical studies of bootstrap variability on large fingerprint datasets and the number of bootstrap replications are explored in Sec. 5. The general formulas of hypothesis testing along with an algorithm for computing the correlation coefficient in our applications are provided in Sec. 6. The results of examples involving both high- and low-accuracy algorithms^1^ are shown in Sec. 7. Finally, the conclusions and discussion can be found in Sec. 8.

## 2. The Formulations of Discrete Probability Distributions of Similarity Scores and ROC Curve

Without loss of generality, the similarity scores used by a matching algorithm are expressed inclusively using the integer score set {*s*} = {*s*_min_, *s*_min_+ 1, …, *s*_max_}. Let **G** denote N_G_ genuine scores generated by comparing two different images of the same subject and **I** denote N_I_ impostor scores created by matching two images of two different subjects.

Some scores in {*s*} may very well be used multiple times in **G** and/or **I**, and some may not be used at all. Hence, let *P_i_*(*s*), where *s*_min_ ≤ *s* ≤ *s*_max_ and *i* ∈ {G, I}, denote the empirical probabilities of the genuine scores and the impostor scores at a score *s*, respectively. Certainly, both of them are normalized, i.e., $\sum_{\tau =s\min}^{s\max}P_{i}(\tau )=1$, where i ∈ {G, I}.

The cumulative discrete probability distribution functions of genuine scores and impostor scores are defined in this article to be the probabilities cumulated from the highest score *s*_max_ down to the integer score *s*. Thus, the cumulative probabilities of genuine scores and impostor scores, i.e., the *TAR* and *FAR*, respectively, are expressed as

(1) $$C_{i}(s)=\sum_{\tau =s}^{s\max}P_{i}(\tau )$$

where *s*_min_ ≤ *s* ≤ *s*_max_ and *i* ∈ {G, I}.

It is assumed that an ROC curve discussed in this article is formed using the trapezoidal rule. Hence, an ROC curve is a curve connecting *s*_max_ − *s*_min_ + 1 points {(*C*_I_(*s*), *C*_G_(*s*)) | *s* = *s*_max_, *s*_max_−1, …, *s*_min_} using line segment in the *FAR*-and-*TAR* coordinate system, and extending to the origin of the coordinate system. Overlap of points (*C*_I_(*s*), *C*_G_(*s*)) can occur, when both *P*_I_(*s*) and *P*_G_(*s*) are zero. An ROC curve goes horizontally, vertically, or inclined upper-rightwards at a score *s*, depending on whether only *P*_I_(*s*) is nonzero, or only *P*_G_(*s*) is nonzero, or both of them are nonzero, respectively.

## 3. Methods of Computing Measures

### 3.1 Scenario 1: The Estimated *TAR* at a Specified *FAR*

Given a *FAR* = *f* where 0 < *f* < 1, without loss of generality, the corresponding threshold score *t* is defined to satisfy

(2) $$C_{1}(t+1)<f\;\text{and}\;C_{1}(t)\geq f,$$

where both *t* and (*t* + 1) ∈ {*s*}. Hence, *P*_I_(*t*) = *C*_I_(*t*) − *C*_I_(*t* + 1) > 0, i.e., the probability of impostor scores at the threshold score *t* is always positive in Scenario 1.

It was shown in Ref. [5] that by using ROC curve the estimated *TAR* at a specified *FAR* = *f* is given by

(3) $$T\hat{A}R(f)=C_{G}(t+1)+P_{G}(t)\times \frac{f-C_{1}(t+1)}{P_{I}(t)}.$$

This formula takes into account the ties of genuine scores and impostor scores, which not only can often occur but also can be large while dealing with large size of datasets.

### 3.2 Scenario 2: The Estimated *TAR* and *FAR* at a Given Threshold

The estimated *TAR* and *FAR* at a given threshold score *t* (*t* might not be a legitimate score) are expressed by

(4) $$\begin{array}{c} T\hat{A}R(t)=C_{G}(s)\\ F\hat{A}R(t)=C_{I}(s) \end{array}\text{for}\;t\in (s-1,s]\;\text{and}\;s_{\min}\leq s\leq s_{\max}.$$

In other words, the probabilities are cumulated from the highest similarity score down to the legitimate integer score that is the ceiling of the input threshold score *t* [25].

### 3.3 Scenario 3: The Estimated *EER*

Generally speaking for discrete probability distribution functions there might not exist such a similarity score (range) at which the probability of type I error denoted by *ER_I* is exactly equal to the probability of type II error denoted by *ER_II*. At a similarity score *s*∈{*s*}, their estimators are expressed as

(5) $$\begin{array}{l} E\hat{R}\text{\_}I(s)=1-C_{G}(s+1)\\ E\hat{R}\text{\_}II(s)=C_{I}(s) \end{array}\text{for}\;s_{\min}\leq s\leq s_{\max},$$

where *C*_G_(*s*_max_ + 1) = 0 is assumed [25].

As the score *s* runs from the highest score *s*_max_ down to the lowest score *s*_min_, the estimator $E\hat{R}\text{\_}I(s)$ decreases from 1 to *P*_G_(*s*_min_), but the estimator $E\hat{R}\text{\_}II(s)$ increases from *P*_I_(*s*_max_) to 1. Both of them are step functions. Hence, the absolute difference $\left|E\hat{R}\text{\_}I(s)-E\hat{R}\text{\_}II(s)\right|$ decreases first, and then increases after reaching its minimum. It seems that for discrete distributions the minimum can rarely reach zero. Assume that the minimum is reached when the score *s* is in the range [*s*_1_, *s*_2_]. Then, the estimated *EER* is defined to be

(6) $$E\hat{E}R=\frac{E\hat{R}\text{\_}I(s)+E\hat{R}\text{\_}II(s)}{2}\text{for}\;s\in [s_{1},s_{2}].$$

Since $\left|E\hat{R}\text{\_}I(s)-E\hat{R}\text{\_}II(s)\right|$ has the same minimum value in the range [*s*_1_, *s*_2_], the corresponding threshold score can simply be defined to be

(7) $$T\hat{H}S=\left\lfloor \frac{s_{1}+s_{2}}{2}\right\rfloor .$$

## 4. Methods of Computing Uncertainties —the Nonparametric Two-Sample Bootstrap

The nonparametric two-sample bootstrap [7–10] is employed to compute the estimates of measurement uncertainties in all three scenarios. The algorithm is as follows.

**Algorithm I:** (Nonparametric two-sample bootstrap)

1:	**for** *i* = 1 **to** *B* **do**
2:	select N_G_ scores randomly WR from **G** to form a set {new N_G_ genuine scores}*_i_*
3:	select N_I_ scores randomly WR from **I** to form a set {new N_I_ impostor scores}*_i_*
4:	{new N_G_ genuine scores}*_i_* & {new N_I_ impostor scores}*_i_* = > statistics ${\hat{T}}_{i}^{k}$, *k* =1 or 1, 2
5:	**end for**
6:	$\{{\hat{T}}_{i}^{k}|i=1,\ldots ,B\}\Rightarrow S{\hat{E}}_{B}^{k}$ and ( ${\hat{Q}}_{B}^{k}(\alpha /2)$, ${\hat{Q}}_{B}^{k}(1-\alpha /2)$) where *k* =1,2
7:	**end**

where *B* is the number of two-sample bootstrap replications and WR stands for “with replacement.” The original genuine score set **G** and the original impostor score set **I** are defined in Sec. 2. As shown from Step 1 to 5, Algorithm I runs *B* times. In the *i*-th iteration, N_G_ scores are randomly selected WR from the original genuine score set **G** to form a new set of N_G_ genuine scores, N_I_ scores are randomly selected WR from the original impostor score set **I** to form a new set of N_I_ impostor scores, and then from these two new sets of similarity scores the *i*-th bootstrap replications of the estimated statistics of interest, i.e., ${\hat{T}}_{i}^{k}$, *k* = 1 or 1, 2, are generated.

The number *k* depends on the scenario. While *FAR* is specified, ${\hat{T}}_{i}^{l}$ stands for the *i*-th bootstrap replication of the estimated *TÂR*(*f*) derived using Eq. (3). If the threshold score *t* is given, ${\hat{T}}_{i}^{l}$ is the *i*-th replication of the estimated *TÂR*(*f*) and ${\hat{T}}_{i}^{2}$ is the *i*-th replication of the estimated *FÂR*(*t*) derived using Eq. (4). When the *EER* is the statistic of interest, ${\hat{T}}_{i}^{l}$ is the *i*-th replication of the estimated *EÊR* obtained using Eq. (6).

Finally as indicated in Step 6, from the sets $\left\{{\hat{T}}_{i}^{k}|i=1,\ldots ,B\right\}$, *k* = 1 or 1, 2, the standard error $S{\hat{E}}_{B}^{k}$ estimated by the sample standard deviation of the *B* replications, and the estimators of the *α*/2 100 % and (1−*α*/2) 100 % quantiles of the bootstrap distribution, denoted by ${\hat{Q}}_{B}^{k}(\alpha /2)$ and ${\hat{Q}}_{B}^{k}(1-\alpha /2)$, at the significance level *α* can be calculated [10]. The Definition 2 of quantile in Ref. [26] is adopted. That is, the sample quantile is obtained by inverting the empirical distribution function with averaging at discontinuities. Thus, ( ${\hat{Q}}_{B}^{k}(\alpha /2)$, ${\hat{Q}}_{B}^{k}(1-\alpha /2)$) stands for the estimated bootstrap (1−*α*) 100 % CÎ. If 95 % CÎ is of interest, then *α* is set to be 0.05.

If the statistic of interest is normally distributed, then the estimated 95 % CÎ can also be computed using the estimated SÊ. On the other hand, if these two types of 95 % CIs for the statistic of interest match well, then it indicates that the distribution of the statistic of interest is normal, as stated in Sec. 1.

## 5. Empirical Studies of Bootstrap Variability and the Number of Bootstrap Replications

### 5.1 Variability of Two-Sample Bootstrap Estimates

As discussed in the literature [8–10], bootstrap estimates can have substantial variance that comes from two distinct sources: sampling variability and bootstrap resampling variability; and the bootstrap variance results in the variability of the SE as well as of the lower and upper bounds of CI of the bootstrap distribution of the statistic of interest. Hence, the sample size and the number of bootstrap replications can be determined by studying the variances of SE and of the two bounds of CI of the bootstrap distribution.

As stated in Sec. 1, the issue of sample sizes, i.e., both N_G_ and N_I_ in the context of fingerprint-image matching algorithms, was studied [15]. Thus, they are fixed throughout the computation in this article. However, as discussed in Sec. 1, the number of two-sample bootstrap replications *B* needs to be investigated for our applications.

### 5.2 Compute Coefficients of Variation

The empirical studies of bootstrap variability were carried out on different statistics of interest in all three scenarios as well as on the metric *AURC* [27]. It was found they behaved in the same way. Thus, only the results regarding the statistic of interest *TAR* at a given *FAR* are presented. To take into account the impact of the mean value, the coefficient of variation (CV) is used. Here is an algorithm of computing CVs of SE, lower and upper bounds of CI for *TAR* at a given *FAR*.

**Algorithm II:** (Bootstrap variability)

1:	**for** *i* = 1 **to** *L* **do**
2:	**for** *j* = 1 **to** *B* **do**
3:	select N_G_ scores randomly WR from **G** to form a set {new N_G_ genuine scores}*_j_*
4:	select N_I_ scores randomly WR from **I** to form a set {new N_I_ impostor scores}*_j_*
5:	{new N_G_ genuine scores}*_j_* & {new N_I_ impostor scores}*_j_* => statistic *TÂR_j_*(*f*)*_i_*, as *FAR* = *f*
6:	**end for**
7:	{*TÂR_j_* (*f*)*_i_* | *j* =1,…, *B*} ⇒ *SÊ_B_* (*f*)*_i_*, ${\hat{Q}}_{B}{\left(a/2,f\right)}_{i}$, ${\hat{Q}}_{B}{\left(1-\alpha /2,f\right)}_{i}$
8:	**end for**
9:	$\left\{S{\hat{E}}_{B}{\left(f\right)}_{i},{\hat{Q}}_{B}{\left(a/2,f\right)}_{i},{\hat{Q}}_{B}{\left(1-\alpha /2,f\right)}_{i}|i=1,\ldots ,L\}\Rightarrow {C\hat{V}}_{B,L}(\kappa \right)$, *κ* = **SE***_B,L_* **(*f*)**, **Q***_B,L_* **(*α*/2**, ***f***), *or* **Q***_B,L_* (**1** −***α*/2**, ***f***)
10:	**end**

where *L* is the number of Monte Carlo iterations and *B* is the number of bootstrap replications. As indicated from Step 1 to 8, Algorithm II runs *L* iterations for a specified *B*. The part from Step 2 to 7 is equivalent to the nonparametric two-sample bootstrap Algorithm I, which generates the *i*-th *SÊ_B_*(*f*)*_i_*, ${\hat{Q}}_{B}{\left(a/2,f\right)}_{i}$ and ${\hat{Q}}_{B}{\left(1-\alpha /2,f\right)}_{i}$ in the *i*-th iteration for a specified *B*.

As shown in Step 9, for a specified *B*, after *L* iterations of executing two-sample bootstrap algorithm, the following three sets are generated,

(8) $$\begin{array}{l} SE_{B,L}(f)=\{S{\hat{E}}_{B}{\left(f\right)}_{i}|i=1,\ldots ,L\},\\ Q_{B,L}(\alpha /2,f)=\{{\hat{Q}}_{B}(\alpha /2,f)i|i=1,\ldots ,L\},\\ Q_{B,L}(1-\alpha /2,f)=\{{\hat{Q}}_{B}(1-\alpha /2,f)i|i=1,\ldots ,L\}. \end{array}$$

Thereafter, from these three sets, three CVs of SE, lower bound and upper bound of CI, can be obtained, respectively,

(9) $$\begin{array}{c} C{\hat{V}}_{B,L}(\kappa )=\sqrt{\frac{{V\hat{A}R}_{B,L}(\kappa )}{{\hat{E}}_{B,L}(\kappa )}},\\ \kappa =SE_{B,L}(f),Q_{B,L}(\alpha /2,f),or\;Q_{B,L}(1-\alpha /2,f), \end{array}$$

where VÂR*_B,L_*(*κ*) denotes variance and Ê*_B,L_*(*κ*) denotes mean of the set of *L* values. It is clear that the three CVs are functions of *B* and *L*, besides the significance level *α* and the *FAR f*. Therefore, the number of bootstrap replications *B* can be determined by the tolerable CVs. Then, the question is: How many iterations *L* are sufficient for a specified *B* to guarantee the accuracy of the Monte Carlo computation?

### 5.3 The Number of Monte Carlo Iterations and Results of Coefficients of Variation

Two fingerprint-image matching algorithms, high-accuracy A1 and low-accuracy A2, were employed. The significance level *α* was set to be 5 % and the *FAR* was specified at 0.001. The estimates of CVs of SE, lower bound and upper bound of 95 % CI are denoted by $C\hat{V}\mathrm{SE}$, $C\hat{V}\mathrm{LB}$, and $C\hat{V}\mathrm{UB}$, respectively. The empirical bootstrap variability studies consume tremendous CPU time. In order to save execution time and in the meantime to preserve the computation accuracies, an approach of numerical analysis rather than statistical analysis is carried out in the following.

For high-accuracy Algorithm A1, the number of replications B was first set to be from 200 up to 1000 at intervals of 200. For each B, the number of Monte Carlo iterations L ran from 100 up to 1000 at intervals of 100, and thus 10 estimates of CVSEs, CVLBs, and CVUBs were generated. The minimum, maximum, and range of these 10 estimates in each case are shown in Table 1.

It is observed from Table 1 that the maximum $C\hat{V}\mathrm{SEs}$ get smaller as *B* increases and the ranges of 10 estimated CVSEs change from about 0.007 down to 0.002; the maximum $C\hat{V}\mathrm{LBs}$ and $C\hat{V}\text{UBs}$ are less than 0.00007 and the ranges are not greater than 0.000008. Therefore, the number of required Monte Carlo iterations *L* does not need to vary from 100 up to 1000 at intervals of 100. For estimating CVs, as the number of replications *B* varied from 1200 up to 2000 at intervals of 200, *L* was set to be 500. The corresponding estimates of CVs are shown in Table 2.

As shown in Table 3 for low-accuracy Algorithm A2, which has the same structure as Table 1, the ranges of 10 estimated CVSEs vary from about 0.006 down to 0.003. The maximum $C\hat{V}\text{LBs}$ and $C\hat{V}\text{UBs}$ are less than 0.0012, and the ranges are less than 0.0002. Thus, the number of iterations *L* can also be set at 500. This is how Table 4 was created for Algorithm A2 for the number of replications *B* greater than 1000.

The CVs for low-accuracy Algorithm A2 are all greater than those for high-accuracy Algorithm A1, correspondingly. This is consistent with what was learned before [3, 4, 15]. Hence, the tolerances for low-accuracy algorithms should be set larger than those for high-accuracy algorithms if necessary.

### 5.4 Tolerances for the Coefficients of Variation

A further investigation was taken on the three CVs of Algorithms A1 and A2, generated by 500 Monte Carlo iterations with 2000 bootstrap replications, respectively, which are listed in the last column of Table 2 and Table 4. For each algorithm, 500 Monte Carlo iterations generate 500 estimated SEs, lower bounds, and upper bounds of 95 % CIs, respectively, which form distributions as indicated in Eq. (8). From each of these six distributions, the estimated mean, SE, CV, and 95 % CI were computed and shown in Table 5. Certainly, the estimated CVs in Table 5 are the same as those in Table 2 and Table 4, respectively.

It is demonstrated in Table 5 that the distribution of SÊs is of less dispersion than the distributions of estimated lower bounds and upper bounds of 95 % CIs, respectively, regardless of the accuracy of the algorithm. This is because in the tail of the distribution fewer samples occur [10]. However, the means of SÊs are much less than 1, and on the contrary the means of two estimated bounds of 95 % CIs are very close to 1 for high-accuracy algorithm and quite close to 1 for low-accuracy algorithm. This is why the $C\hat{V}$ of SÊ is much larger than the $C\hat{V}s$ of two estimated bounds of 95 % CIs for each algorithm. As a consequence, the tolerance for CV of SE needs to be set larger than those for CVs of two bounds of 95 % CIs.

### 5.5 The Number of Bootstrap Replications

**Fig. 3:**
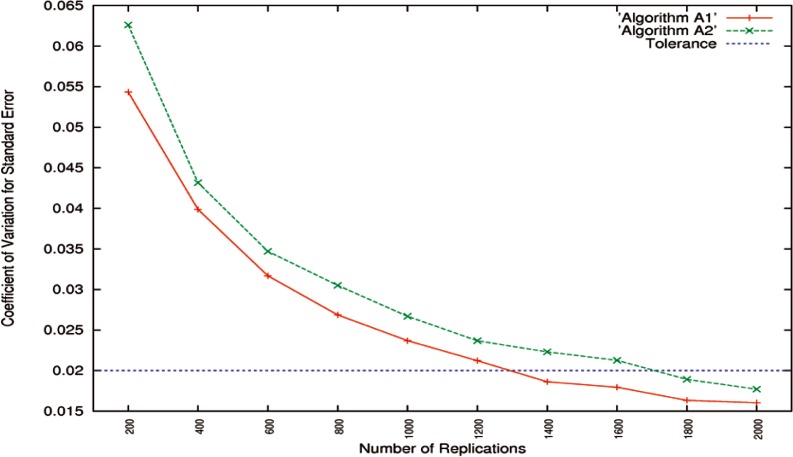
$C\hat{V}\text{SEs}$ for high-accuracy Algorithm A1 and low-accuracy Algorithm A2 as a function of the number of replications. The tolerance is set to be 0.02.

All $C\hat{V}\text{SEs}$, $C\hat{V}\text{LBs}$, and $C\hat{V}\text{UBs}$ of Algorithms A1 and A2 from Table 1 to Table 4 are depicted in Fig. 3 through Fig. 5. In the cases where the number of replications *B* was set to be from 200 up to 1000 at intervals of 200, only the maximum $C\hat{V}\text{SEs}$, $C\hat{V}\text{LBs}$, and $C\hat{V}\text{UBs}$ from Table 1 and Table 3 are used.

The $C\hat{V}\text{SEs}$ of Algorithms A1 and A2 are drawn in Fig. 3. It shows that all $C\hat{V}\text{SEs}$ decrease as the number of replications *B* increases. If the tolerance is set to be 0.02, 1400 two-sample bootstrap replications are sufficient for high-accuracy Algorithm A1, and 1800 replications are enough for low-accuracy Algorithm A2. To achieve the same level of accuracy, high-accuracy matching algorithms generally require less execution than low-accuracy algorithms do [3, 4, 15].

**Fig. 4:**
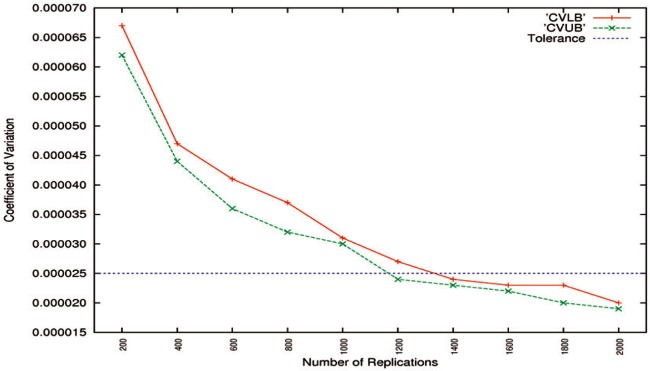
$C\hat{V}\text{LBs}$ and $C\hat{V}\text{UBs}$ for high-accuracy Algorithm A1 as a function of the number of replications. The tolerance is set to be 0.000025.

**Fig. 5:**
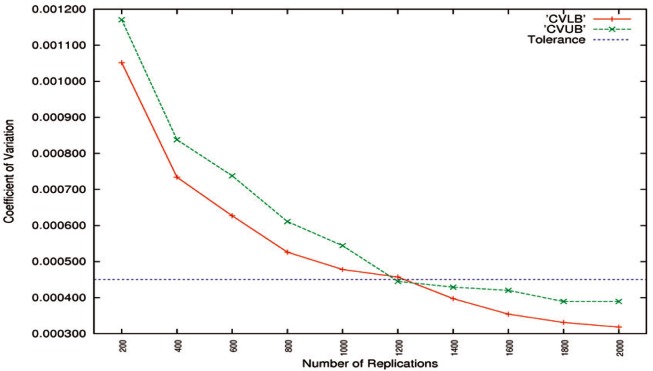
$C\hat{V}\text{LBs}$ and $C\hat{V}\text{UBs}$ for low-accuracy Algorithm A2 as a function of the number of replications. The tolerance is set to be 0.000450.

The $C\hat{V}\text{LBs}$ and $C\hat{V}\text{UBs}$ for Algorithm A1 are shown in Fig. 4. As discussed in Sec. 5.4, the tolerances for CVs of two bounds of 95 % CIs should be set smaller. Hence, if the tolerance is set to be 0.000025, 1400 replications can meet the requirement. Those for Algorithm A2 are depicted in Fig. 5. As pointed out in Sec. 5.3, the tolerance for low-accuracy algorithms should be set larger. Thus, if the tolerance is set to be 0.000450, 1400 replications can satisfy the restriction.

The maximum tolerance set for CVs so far is 0.02, which is acceptable [10]. This 2 % tolerance holds good not only for the statistic of interest in Scenario 1, but also for the statistics of interest in Scenarios 2 and 3 as well as for the metric *AURC*, as stated in Sec. 5.2 [27]. To reconcile numbers of replications for different qualities of algorithms, and further to be more conservative, it is suggested that 2000 two-sample bootstrap replications be required in order to achieve statistical accuracy of computation.

## 6. Hypothesis Testing

From comparison perspective, the statistics *TAR* at a given *FAR* and *EER* are of interest, as pointed out in Sec. 1. In all cases encountered in the references [5, 6] and Sec. 7.1, it was found that the 95 % CIs of the statistics of interest computed using the definition of quantile did match the 95 % CIs calculated if the distributions of the 2000 bootstrap replications of the statistics were assumed to be normal. The matching was up to the third to fourth decimal place. The higher the accuracy of algorithm is, the more decimal places are matched. Moreover, the Shapiro-Wilk normality test [12] was conducted on the 2000 bootstrap replications of the statistics of interest. It was observed that the majority of *p*-values were greater than 5 %, especially for high-accuracy algorithms. As a result, it is suggested that the statistics of interest in our applications be assumed to be normally distributed regardless of the discrete empirical distributions of genuine and impostor scores.

Under the normality assumption, in analogy to *AURC* [3, and references therein], the straightforward way to perform the significance test is the *Z*-test. The *Z* statistic has the standard normal distribution with zero expectation and a variance of one. The SEs of all statistics of interest involved in the *Z* statistic in our applications can be computed using the nonparametric two-sample bootstrap.

There is no reason to believe *a priori* that the performance of one algorithm is likely to be better than a hypothesized value or the performance of the other algorithm. Further, the two-tailed test is generally more conservative than the one-tailed test in the sense that the former is more difficult to reject the null hypothesis for a given significance level [28]. Thus, the two-tailed test is used in this article. In the case that the alternative hypothesis is accepted, the sign of the difference is employed to determine which is better than the other.

### 6.1 One-Algorithm Hypothesis Testing

Let *T* denote a probability measure, such as *TAR* and *EER*, for an algorithm and *μ*_o_ denote the hypothesized value. Then, the null and alternative hypotheses are

(10) $$\begin{array}{l} H_{o}:T=\mu_{o}\\ H_{a}:T\neq \mu_{o}. \end{array}$$

Based on the normality assumption, the *Z* statistic is

(11) $$Z=\frac{\hat{T}-\mu_{o}}{\mathrm{SE}(\hat{T})}$$

where $\hat{T}$ is the estimator of the statistic of interest and $\mathrm{SE}(\hat{T})$ stands for its SE.

While evaluating the performance of an algorithm, besides *p*-value, other factors also need to be taken into account, such as the characteristic of the statistic of interest (the larger the better or the smaller the better) and the sign of the difference between the estimator and the accuracy criterion value. For instance, if the statistic of interest is *TAR* (the larger the better) and its estimator is less than *μ*_o_, then less-than-5 % *p*-value indicates that this algorithm fails the test.

### 6.2 Two-Algorithm Hypothesis Testing

Let *T*_1_ and *T*_2_ denote the probability measures, such as *TAR* and *EER*, for Algorithms 1 and 2, respectively. Then, the null and alternative hypotheses are

(12) $$\begin{array}{l} H_{o}:T_{1}=T_{2}\\ H_{a}:T_{1}\neq T_{2}. \end{array}$$

Based on the normality assumption, the general *Z* statistic for two-algorithm hypothesis testing is expressed as

(13) $$Z=\frac{{\hat{T}}_{1}-{\hat{T}}_{2}}{\sqrt{{\mathrm{SE}}^{2}({\hat{T}}_{1})+{\mathrm{SE}}^{2}({\hat{T}}_{2})-2\;r\mathrm{SE}({\hat{T}}_{1})\mathrm{SE}({\hat{T}}_{2})}}$$

where ${\hat{T}}_{1}$ and ${\hat{T}}_{2}$ are two estimators of the statistics of interest, $\mathrm{SE}({\hat{T}}_{1})$ and $\mathrm{SE}({\hat{T}}_{2})$ stand for their SEs, respectively, and *r* is the correlation coefficient between ${\hat{T}}_{1}$ and ${\hat{T}}_{2}$. If the two statistics of interest are positively correlated and the correlation coefficient *r* is not taken into account, it can leave the denominator of Eq. (13) larger and the *Z* score smaller; thereby reduce the chance of detecting a difference between the performances of two algorithms.

### 6.3 An Algorithm for Computing the Correlation Coefficient

The two statistics of interest of any two algorithms may or may not be correlated, depending on how the sets of similarity scores are generated. In our tests, different fingerprint-image matching algorithms generated different sets of similarity scores, respectively, using the same set of fingerprint images. Any two scores with the same ordinal number of entry in the two sets of similarity scores were generated using the same two images, and thus co-varied. All algorithms have the same tendency to assign a higher (or lower) similarity score to the match where two fingerprint images are more (or less) similar. Such a characteristic may cause positive correlation between two sets of similarity scores of two algorithms. Subsequently, it may result in the positive correlation between the statistics of interest of two algorithms.

It is assumed that any two Algorithms denoted by A and B generate the same amount of genuine scores as well as impostor scores. The genuine score sets and the impostor score sets of Algorithms A and B are denoted, respectively, by **G***^i^* and **I***^i^* where *i* ∈ {A, B}, in analogy to **G** and **I** defined in Sec. 2. The two *j*-th genuine (impostor) scores, say, $m_{j}^{i}(n_{j}^{i})$ where *i* ∈ {A, B}, co-vary. An algorithm for computing the correlation coefficient of the statistic of interest *T*, i.e., either *TAR* or *EER*, is as follows.

**Algorithm III:** (Correlation coefficient)

1:	**for** *i* = 1 **to** *M* **do**
2:	Synchronized_WR_Random_Sampling (N_G_, **G^A^**, Θ**^A^***_i_*, **G^B^**, Θ**^B^***_i_*)
3:	Synchronized_WR_Random_Sampling (N_I_, **I^A^**, Ξ**^A^***_i_*, **I^B^**, Ξ**^B^***_i_*)
4:	the new genuine score set Θ**^A^***_i_* and the new impostor score set Ξ**^A^***_i_*= > statistic ${\hat{T}}_{i}^{A}$
5:	the new genuine score set Θ**^B^***_i_* and the new impostor score set Ξ**^B^***_i_* = > statistic ${\hat{T}}_{i}^{B}$
6:	**end for**
7:	$\left\{{\hat{T}}_{i}^{A}|i=1,\ldots ,M\right\}$ and $\left\{{\hat{T}}_{i}^{B}|i=1,\ldots ,M\right\}$ => the correlation coefficient *r*^AB^*_T_*
8:	**end**
1.1:	**function** Synchronized_WR_Random_Sampling (*N*, **S^A^**, Γ**^A^**, **S^B^**, Γ**^B^**)
1.2:	**for** *j* = 1 **to** *N* **do**
1.3:	select randomly WR an index *k* ∈ {1, …, *N*}
1.4:	*γ*^A^*_j_* = *s*^A^*_k_*
1.5:	*γ*^B^*_j_* = *s*^B^*_k_*
1.6:	**end for**
1.7:	**end function**

where *s*^A^*_k_*, *γ* ^A^*_j_*, *s*^B^*_k_*, and *γ*^B^*_j_* are members of the score sets **S^A^**, **Γ^A^**, **S^B^**, and **Γ^B^** respectively. Based on our bootstrap variability studies, the number of iterations *M* is set to be 2000.

From Step 1 to 6, this algorithm runs M iterations. In the i-th iteration, the synchronized WR random sampling is carried out on **G^A^** and **G^B^** (**I^A^** and **I^B^**) to generate two new genuine (impostor) score sets Θ**^A^***_i_* and Θ**^B^***_i_* (Ξ**^A^***_i_* and Ξ**^B^***_i_*), respectively. From Step 1.1 to 1.7, during the sampling iterations, if a similarity score of Algorithm A is randomly selected, then the co-varying similarity score (i.e., with the same ordinal number of entry) of Algorithm B is also selected. All correlated similarity scores between two algorithms are randomly selected simultaneously. Hence, the correlation in the similarity scores between two algorithms is preserved if there is any. After sampling, in Step 4 (5), the *i*-th estimated statistic ${\hat{T}}_{i}^{A}\;({\hat{T}}^{B}{}_{i})$ of Algorithm A (B) is computed from the new score sets Θ**^A^***_i_* and Ξ**^A^***_i_* (Θ**^B^***_i_* and Ξ**^B^***_i_*). Finally after *M* iterations in Step 7, the correlation coefficient *r*^AB^*_T_* of the statistic of interest *T* of Algorithms A and B can be calculated from the two sets of estimated statistics of interest.

This algorithm involves a synchronized random sampling. In practice, if the *p*-value is not considerably different from the critical values, such as 5 %, 1 %, etc., then in order to reduce the computational fluctuation this algorithm needs to run multiple times. Even though the fluctuation is quite small based on our observations in our test, to be more conservative, in this article, the average out of 10 runs was taken to be the resultant correlation coefficient for significance test.

As stated in Sec. 6.2, Eq. (13) is the general formula for performing two-algorithm hypothesis testing. If the method of generating similarity scores as described in this section is encountered, then two co-varied scores with the same ordinal number of entry in the two sets of similarity scores of two algorithms can be treated as a score pair as indicated in the synchronized random sampling in Algorithm III, and the bootstrap objects can be such correlated pairs of similarity scores. Thus, to perform hypothesis testing, Eq. (11) can be employed. However, after expansion the expression of SE in the denominator in Eq. (11) is the same as the denominator in Eq. (13). To explicitly illustrate the correlation of two statistics of interest in our applications, the correlation coefficients will be calculated. Thus, Eq. (13) will be employed for two-algorithm hypothesis testing.

## 7. Results

Algorithms A1 and A2 used for empirical studies of bootstrap variability in Sec. 5 were also taken to be examples for measures and uncertainties. Five algorithms, B1 through B5, were used as examples for evaluations and comparisons. Algorithms A1 and B1 are the same one. B1 and B2 are of high accuracy; B3 through B5 are of relatively low accuracy; and A2 is of low accuracy. More examples can be found in Ref. [5, 6, 29]. Different algorithms employed different types of scoring systems, such as integers, real numbers in different ranges. Results were kept up to six decimal places for illustration.

### 7.1 Measures and Uncertainties

#### 7.1.1 Measures and Uncertainties of *TAR* at a Specified *FAR*

The estimated *TÂR* (*f*) at a specified *FAR* can be computed using Eq. (3). The *FAR* was set to be 0.001 [4, 15]. In Table 6 are shown the estimates of *TAR*s, SEs, and 95 % CIs for high-accuracy A1 and low-accuracy A2. As indicated in Sec. 4, the 95 % CIs were calculated using the Definition 2 of quantile in Ref. [26]. The 95 % CIs can also be computed if the distribution of 2000 bootstrap replications of the statistic *TÂR* (*f*) for each algorithm is assumed to be normal. These two types of 95 % CIs do match up to the third to fourth decimal place depending on the accuracy of the algorithm. For example, for high-accuracy Algorithm A1, the 95 % CI of the estimated *TÂR (f*) is (0.992622, 0.993922) as shown in Table 6, and the 95 % CI assuming normal distribution is (0.992618, 0.993892) using the estimated SÊ 0.000325. It is also found that the higher the accuracy of the algorithm is, the smaller the SE is. These observations are consistent with those in Ref. [3, 15].

As investigated in Sec. 5.4, for Algorithms A1 and A2, the nonparametric two-sample bootstrap was executed for 500 times while the number of bootstrap replications *B* was fixed at 2000. The resultant 95 % CIs of 500 SEs, lower bounds and upper bounds of 95 % CIs for A1 and A2, respectively, were shown in the last column of Table 5. Note that the results shown in Table 6 were generated only by a random run that is not one of the above 500 runs. However, it is observed that the SEs, lower bounds and upper bounds of 95 % CIs for A1 and A2 shown in Table 6 all fall in the corresponding 95 % CIs shown in Table 5.

This observation demonstrates that although computing measurement uncertainties using two-sample bootstrap is a stochastic process, the SE, lower bound and upper bound of 95 % CI of the statistic of interest may fall into the CIs with 95 % probability, which are generated by many executions of two-sample bootstrap with 2000 bootstrap replications. Moreover, these CIs are very narrow from the practical point of view.

#### 7.1.2 Measures and Uncertainties of *TAR* and *FAR* at a Given Threshold

In Table 7 are shown the estimates of *TAR*s and *FAR*s along with their estimated SEs and 95 % CIs for Algorithms A1 and A2 while the threshold score *t* is given. The 95 % CIs shown in Table 7, which were computed using the definition of quantile, do match the 95 % CIs up to the third to fourth decimal place depending on the accuracy of the algorithm for both *TAR*s and *FAR*s, which were calculated if the distributions of 2000 bootstrap replications of the statistics *TÂR* (*t*) and *FÂR* (*t*) are assumed to be normal, respectively. For instance, for high-accuracy Algorithm A1, the 95 % CI of the estimated *FÂR* (*t*) is (0.000820, 0.001184) as shown in Table 7 and the 95 % CI assuming normal distribution is (0.000830, 0.001186) using the estimated SÊ 0.000091.

The input threshold can vary. To show the operational significance, the estimated threshold score derived from Eq. (2) at a given *FAR* 0.001 in Sec. 7.1.1 was chosen to be the input threshold score *t* for each algorithm in Table 7 [6]. It is observed that for each algorithm the estimated statistic of interest *TÂR* (*t*) and the specified *FAR* 0.001 in Table 6 all fall into the corresponding 95 % CIs in Table 7, and reversely so does the estimated *TÂR* (*t*) in Table 7. Moreover, all corresponding 95 % CIs in these two tables are equivalent especially for high-accuracy algorithm. All these observations indicate that the computation using the nonparametric two-sample bootstrap with 2000 bootstrap replications is quite self-consistent.

The two 95 % CIs of the estimated *FÂR* (*t*) and *TÂR* (*t*), formed by 2000 *FAR*-and-*TAR* points paired by bootstrap replications, constitute a rectangle around the estimators. If the threshold changes, the rectangle can move along an ROC curve. The bootstrap replications of *FAR* are not correlated with the bootstrap replications of *TAR* at any threshold. By no means, this rectangle is a 95 % confidence rectangle. The rectangle only shows the bounds of the two 95 % CIs [6].

#### 7.1.3 Measures and Uncertainties of *EER*

Besides statistical (random) error, the accuracy of *EER* also includes systematic error stemming from the discreteness of the distributions of similarity scores, which is expressed in terms of the relative error, i.e., half of the minimum of the absolute difference $\left|E\hat{R}\text{\_}I(s)-E\hat{R}\text{\_}II(s)\right|$ divided by the estimated *EÊR* derived from Eq. (6). The systematic errors of two algorithms are shown in Table 8. They can reach as high as 0.51 %, which occurs even for high-accuracy Algorithm A1. It is also noticed that the minimum of the absolute difference can occur within a score range rather than at a single score due to the reason stated in Sec. 3.3.

In Table 9 are presented the estimates of *EER*s along with their estimated SEs and 95 % CIs for high-accuracy Algorithm A1 and low-accuracy Algorithm A2. As expected, the higher the accuracy of algorithm is, the smaller the estimated *EÊR* is. This is because the two distributions of genuine scores and impostor scores are more apart and thus the ROC curve is higher [3, 4]. Further, the 95 % CIs shown in Table 9 computed using the definition of quantile do match the 95 % CIs up to the third to fourth decimal place depending on algorithm’s accuracy, which were calculated if the distributions of 2000 bootstrap replications of the statistic *EER* are assumed to be normal. For example, for high-accuracy Algorithm A1, the 95 % CI of the estimated *EÊR* is (0.005511, 0.006703) as shown in Table 9 and the 95 % CI assuming normal distribution is (0.005474, 0.006654) using the estimated SÊ 0.000301.

### 7.2 Evaluations and Comparisons

High-accuracy Algorithms B1 and B2 were taken as examples for one-algorithm hypothesis testing related to evaluations, while *TAR* at a specified *FAR* 0.001 was employed. Relatively low-accuracy Algorithms B3 through B5 were used for two-algorithm significance test related to comparisons, while *EER* was used. The method applied to *TAR* can be applied to *EER*, and vice versa. The only difference is that for *TAR* it is the larger the better, but for *EER* it is the smaller the better. More examples can be found in Ref. [29]. The estimates of *TAR*s, *EER*s, SEs, and 95 % CIs of B1 through B5 are presented in Table 10 and Table 11, respectively.

#### 7.2.1 One-Algorithm Hypothesis Testing

**Fig. 6:**
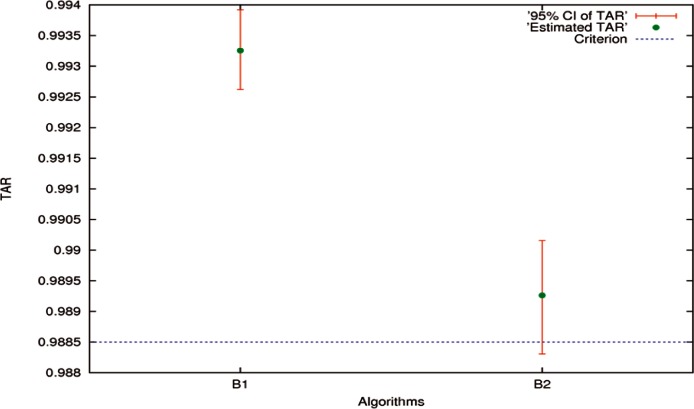
The estimates of *TAR*s and 95 % CIs for Algorithms B1 and B2 at a specified *FAR* 0.001, along with the hypothesized value *μ*_o_ set at 0.988500.

The estimates of *TAR*s and 95 % CIs for Algorithms B1 and B2 are drawn in Fig. 6. For illustration, assume that the hypothesized value *μ*_o_ was set to be 0.988500. By applying Eq. (11), the two-tailed *p*-values were calculated. They are shown in Table 12. For B1, the *p*-value was equal to 0.0000, and thus the alternative hypothesis *H*_a_: *T* ≠ *μ*_o_ is very strongly accepted. With the positive sign of the difference between *TÂR* (*f*) and *μ*_o_, it is concluded that the *TÂR* (*f*) of B1 is very significantly greater than the accuracy criterion value 0.988500. In other words, Algorithm B1 passes the test.

For B2, the two-tailed *p*-value was 0.1049, which was greater than 5 %. It suggests that the null hypothesis *H*_o_ : *T* = *μ*_o_ be accepted. That is, the difference between *TÂR* (*f*) and *μ*_o_ is not real but by chance at the significance level 10 %. Hence, Algorithm B2 fails the test, if the performance is required to be better than the accuracy criterion value *μ*_o_ set as 0.988500.

Indeed, it is trivial to prove that if the 95 % CI contains the hypothesized value *μ*_o_, the null hypothesis *H*_o_ can be accepted with at least 5 % significance level; otherwise, *H*_o_ is rejected with at most 5 % significance level. Both are with respect to the two-tailed hypothesis testing. However, the approach of merely using the relative position between 95 % CI and the horizontal line at the hypothesized value does not provide quantitative information regarding the statistical significance of the difference.

#### 7.2.2 Two-Algorithm Hypothesis Testing

**Fig. 7:**
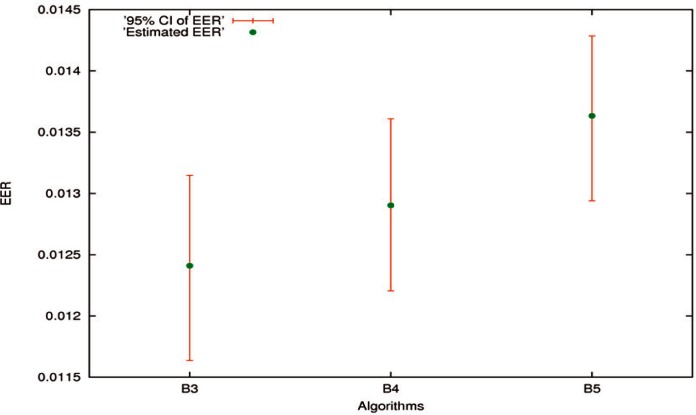
The estimates of *EER*s and 95 % CIs for Algorithms B3 through B5.

The estimates of *EER*s and 95 % CIs for relatively low-accuracy Algorithms B3 through B5 are drawn in Fig. 7. The 95 % CIs of these three algorithms mutually overlap. The hypothesis testing for two algorithms cannot be judged merely using the confidence interval approach.

The average correlation coefficients of *EER* among B3 through B5 out of ten runs using the algorithm in Sec. 6.3 are presented in Table 13. The average correlation coefficient of *EER* between high-accuracy Algorithms B1 and B2 was 0.567842, which is larger than those for relatively low-accuracy Algorithms. In this regard, many more examples can be found in Ref. [29]. It is expected that the tendency of assigning higher (lower) similarity scores to the matching results of more (less) similar images for high-accuracy algorithms is stronger than the tendency for relatively low-accuracy algorithms. These results provide evidence that the synchronized algorithm for computing the correlation coefficient is quite reasonable.

After applying Eq. (13), the two-tailed p-values of EERs among B3 through B5 were calculated. They are presented in Table 14. For Algorithms B3 and B4, it was 0.2370, which was much greater than 5 %. It suggests that the null hypothesis *H*_o_: *T*_1_ = *T*_2_ be accepted. That is to say, the difference between the performances of B3 and B4 is not statistically significant. To some extent, this conclusion is supported by the fact that the 95 % CIs of these two algorithms heavily overlap each other, as illustrated in Fig. 7.

For Algorithms B4 and B5, the two-tailed *p*-value was 0.0457. Without considering the correlation coefficient, it increased to 0.1392. As pointed out in Sec. 6.2, neglecting the positive correlation coefficient can reduce the chance of detecting a difference between the performances of two algorithms. Since 0.0457 is slightly less than 5 %, the alternative hypothesis *H*_a_: *T*_1_ ≠ *T*_2_ is accepted with borderline evidence. Due to the sign of the difference between the two estimated *EER*s, the performance of B4 is reasonably better than the performance of B5, even though the 95 % CI of B4 quite overlaps the 95 % CI of B5 as shown in Fig. 7.

For Algorithms B3 and B5, the two-tailed *p*-value was 0.0019, which was much less than 5 %. It suggests that the alternative hypothesis *H*_a_: *T*_1_ ≠ *T*_2_ be strongly accepted. Because of the sign of the difference between the two estimated *EER*s, the performance of B3 is considerably better than the performance of B5, although their 95 % CIs slightly overlap.

Further, the *p*-value 0.0019 between B3 and B5 is much smaller than the *p*-value 0.0457 between B4 and B5. It indicates that the difference between the performances of B3 and B5 is more statistically significant than the difference between the performances of B4 and B5. To some extent, this conclusion can be supported by the relationship among their 95 % CIs as illustrated in Fig. 7.

## 8. Conclusions and Discussion

The measures in operational ROC analysis, such as *TAR*, *EER*, etc., were computed by taking account of the ties of similarity scores at the threshold. The genuine scores at the threshold determined by a given *FAR* must be divided proportionally according to the trapezoidal rule in order to compute the *TAR* for the given *FAR*.

Concerning *EER*, due to discreteness of distributions of similarity scores, generally speaking the probability of type I error can rarely be exactly equal to the probability of type II error. Hence, the systematic error can occur besides statistical error. For example, for Algorithm A1, the estimated systematic error is 1/2 × 0.000061 / 0.006064 = 0.51 % as shown in Table 8. The estimated total relative error due to both systematic error and statistical error is 0.000301/0.006064 = 4.96 % from Table 9. Thus, the systematic error is estimated to be about 10 % of the total relative error. In all other cases encountered in Ref. [6], algorithms had less systematic errors, smaller total relative errors, and smaller ratios of the systematic errors to the total relative errors. Nonetheless, it must be recognized that systematic error exists when *EER* is employed.

The uncertainties of measures in operational ROC analysis in terms of SE and 95 % CI were computed using the nonparametric two-sample bootstrap method. In our applications, tens and hundreds of thousands of similarity scores are used; our statistics of interest are probabilities such as *TAR*, *FAR*, *EER*, etc., rather than a simple arithmetic mean; and our data samples of similarity scores are not normally distributed. Due to these characteristics, the bootstrap variability was restudied empirically to determine the appropriate number of bootstrap replications in our applications, in order to reduce the bootstrap variance and ensure the accuracy of the computation. The number of bootstrap replications in our applications was determined to be 2000.

As pointed out in Sec. 5.1, the variance of two-sample bootstrap is also caused by sample sizes. If the sizes of similarity scores get larger than what were used here, as stated in Sec. 1, there is little improvement in accuracy. On the other hand, if the sample sizes, for instance, in other biometric applications, are less than the ones dealt with here, the same number of bootstrap replications (2000) can be safely applied. Nonetheless, if the number of bootstrap replications needs to be revisited, the empirical methods for studying the bootstrap variability developed in this article should remain the same.

Regarding operational ROC analysis in our applications, it is important to determine whether the difference between the performance of one algorithm and an accuracy criterion value, or the difference between the performances of two algorithms where the correlation is taken into account is statistically significant. In this regard, such hypothesis testing has not been addressed in the literature.

While conducting comparisons, in some cases the 95 % CIs can be applied to some extent. Nonetheless, the issue of determining quantitatively whether the difference is real or by chance must be dealt with using the significance test, especially when 95 % CIs are overlapped. For instance, as demonstrated in Sec. 7.2.2, all three 95 % CIs were mutually overlapped to a certain degree, but the hypothesis testing showed that the statistical significances of the differences in performances among the three algorithms were quite different accordingly in terms of *p*-values. More examples can be found in Ref. [29].

For such comparison issues, the two statistics of interest, *TAR* at a specified *FAR* and *EER*, are typically employed. They can be treated as normally distributed regardless of the distributions of genuine scores and impostor scores. This assumption is supported by the matches in various cases between two types of 95 % CIs. One is computed using the definition of quantile, and the other is calculated if the distribution of 2000 bootstrap replications of the statistic of interest is assumed to be normal. It is also partly supported by the Shapiro-Wilk normality test.

Under the normality assumption, the *Z*-test can be applied. Involved in the *Z*-test, all the SEs can be computed using the nonparametric two-sample bootstrap with 2000 bootstrap replications. In this article, an algorithm is provided to calculate the correlation coefficient between two statistics of interest of two matching algorithms, under the assumption that for these two algorithms any two scores with the same ordinal number of entry in the two sets of similarity scores were generated using the same two images, as discussed in Sec. 6.3. If the orders in the two score sets changed manually, in other words, if the similarity scores with the same ordinal number did not co-vary, then the correlation coefficients computed using the algorithm in Sec. 6.3 would be close to zero. This also supports the synchronized algorithm for computing the correlation coefficient.

In some literature [30], the false non-match rate (*FNMR*) was employed, which is defined to be 1 – *TAR*. It is trivial to prove that as far as SEs, correlation coefficients, *Z* scores, and *p*-values are concerned, there is no difference between *TAR* and *FNMR*. However, the lower (upper) bound of 95 % CI of *FNMR* is equal to one minus the upper (lower) bound of 95 % CI of *TAR* [5, 6]. For *TAR*, two bounds of 95 % CIs are close to 1 as discussed in Sec. 5.4. Thus, for *FNMR*, they are close to 0 instead. Such a difference can have impact on CVs.

In Table 5, if *TAR* is replaced by *FNMR*, the CVs of SE for Algorithms A1 and A2 remain the same; but the CVs of lower bound and upper bound of 95 % CI were 0.003152 and 0.002687 for A1, and 0.001595 and 0.001196 for A2, respectively. These CVs increased considerably; however they were all less than the tolerance 0.02. Hence, the assertion that the number of two-sample bootstrap replications is 2000 is still valid if *FNMR* is employed. Nonetheless, it needs to point out that *FNMR* has more variability than *TAR* regarding the two bounds of 95 % CI.

While dealing with 1-to-n identification issues, cumulative match characteristic (CMC) analysis is employed. A CMC curve is formed by matching each image in the probe with each image in the gallery. To compute the uncertainty of the identification rate at a rank, the bootstrap method can also be applied. Different schemes of resampling probe and gallery can be proposed. Further, if the distribution of the identification rate at a rank can be assumed to be normal, then the *Z*-test can be used to determine the statistical significance of the difference of identification rates.

## Figures and Tables

**Table 1:** High-accuracy Algorithm A1’s minimum, maximum, and range of 10 estimates of $C\hat{V}\text{SEs}$, $C\hat{V}\text{LBs}$, and $C\hat{V}\text{UBs}$, as the number of iterations *L* ran from 100 up to 1000 at intervals of 100 for each specified *B*. *B* ran from 200 up to 1000 at intervals of 200

Num. of replications *B*		200	400	600	800	1000
	Min.	0.047524	0.034664	0.027754	0.023912	0.021570
$C\hat{V}\mathrm{SE}$	Max.	0.054346	0.039866	0.031685	0.026866	0.023686
	Range	0.006822	0.005202	0.003931	0.002954	0.002116
	Min.	0.000062	0.000044	0.000036	0.000030	0.000026
$C\hat{V}\mathrm{LB}$	Max.	0.000067	0.000047	0.000041	0.000037	0.000031
	Range	0.000005	0.000003	0.000005	0.000007	0.000005
	Min.	0.000054	0.000041	0.000032	0.000030	0.000026
$C\hat{V}\mathrm{UB}$	Max.	0.000062	0.000044	0.000036	0.000032	0.000030
	Range	0.000008	0.000003	0.000004	0.000002	0.000004

**Table 2:** High-accuracy Algorithm A1’s $C\hat{V}\text{SEs}$, $C\hat{V}\text{LBs}$, and $C\hat{V}\text{UBs}$, while *B* ran from 1200 up to 2000 at intervals of 200 as the number of iterations *L* was fixed at 500

Num. of replications *B*	1200	1400	1600	1800	2000
$C\hat{V}\mathrm{SE}$	0.021218	0.018613	0.017951	0.016331	0.016040
$C\hat{V}\mathrm{LB}$	0.000027	0.000024	0.000023	0.000023	0.000020
$C\hat{V}\mathrm{UB}$	0.000024	0.000023	0.000022	0.000020	0.000019

**Table 3:** Low-accuracy Algorithm A2’s minimum, maximum, and range of 10 estimates of $C\hat{V}\text{SEs}$, $C\hat{V}\text{LBs}$, and $C\hat{V}\text{UBs}$, as the number of iterations *L* ran from 100 up to 1000 at intervals of 100 for each specified *B*. *B* ran from 200 up to 1000 at intervals of 200

Num. of replications *B*		200	400	600	800	1000
	Min.	0.056895	0.037193	0.031792	0.026763	0.024033
$C\hat{V}\mathrm{SE}$	Max.	0.062609	0.043167	0.034696	0.030500	0.026695
	Range	0.005714	0.005974	0.002904	0.003737	0.002662
	Min.	0.000941	0.000677	0.000519	0.000473	0.000442
$C\hat{V}\mathrm{LB}$	Max.	0.001052	0.000734	0.000627	0.000526	0.000478
	Range	0.000111	0.000057	0.000108	0.000053	0.000036
	Min.	0.001068	0.000685	0.000637	0.000532	0.000488
$C\hat{V}\mathrm{UB}$	Max.	0.001171	0.000838	0.000738	0.000611	0.000544
	Range	0.000103	0.000153	0.000101	0.000079	0.000056

**Table 4:** Low-accuracy Algorithm A2’s $C\hat{V}\text{SEs}$, $C\hat{V}\text{LBs}$, and $C\hat{V}\text{UBs}$, while *B* ran from 1200 up to 2000 at intervals of 200 as the number of iterations *L* was fixed at 500

Num. of replications *B*	1200	1400	1600	1800	2000
$C\hat{V}\mathrm{SE}$	0.023673	0.022299	0.021272	0.018918	0.017705
$C\hat{V}\mathrm{LB}$	0.000457	0.000397	0.000354	0.000331	0.000318
$C\hat{V}\mathrm{UB}$	0.000445	0.000429	0.000420	0.000389	0.000389

**Table 5:** Means, SÊs, $C\hat{V}s$, and 95 % CÎs of distributions of estimated SEs, lower bounds and upper bounds of 95 % CIs for Algorithms A1 and A2, respectively, generated by 500 iterations with 2000 bootstrap replications

	Algorithm	Mean	SÊ	$C\hat{V}$	95 % Confidence interval
A1	Standard error	0.000331	0.0000053	0.016040	(0.000320, 0.000341)
	Lower bound	0.992617	0.0000198	0.000020	(0.992575, 0.992654)
	Upper bound	0.993913	0.0000192	0.000019	(0.993873, 0.993954)
A2	Standard erro	0.003474	0.0000615	0.017705	(0.003362, 0.003618)
	Lower bound	0.789746	0.0002514	0.000318	(0.789244, 0.790220)
	Upper bound	0.804121	0.0003124	0.000389	(0.803522, 0.804700)

**Table 6:** The estimates of *TAR*s, SEs, and 95 % CIs for high-accuracy Algorithm A1 and low-accuracy Algorithm A2, respectively, while *FAR* was specified at 0.001

Algorithm	*TÂR* (*f*)	SÊ	95 % Confidence interval
A1	0.993255	0.000325	(0.992622, 0.993922)
A2	0.796753	0.003503	(0.789545, 0.803961)

**Table 7:** The estimates of *TAR*s and *FAR*s along with their estimated SEs and 95 % CIs for high-accuracy Algorithm A1 and low-accuracy Algorithm A2, respectively, while the threshold score *t* is given, which was obtained while *FAR* was set to be 0.001 in Sec. 7.1.1.

Algorithm	Threshold score *t*	$\frac{T\hat{A}R(t)}{F\hat{A}R(t)}$	SÊ	95 % Confidence interval
		0.993255	0.000337	(0.992605, 0.993905)
A1	455	0.001008	0.000091	(0.000820, 0.001184)
A2	0.634030	0.796753	0.001590	(0.793641, 0.799792)
		0.001000	0.000092	(0.000836, 0.001189)

**Table 8:** The systematic errors of *EER* for Algorithms A1 and A2. The minimum of the absolute difference can occur either at a score or within a score range

Algorithm	Score (range)	Min $\left(|E\hat{R}\text{\_}I(s)-E\hat{R}\text{\_}II(s)|\right)$	*EÊR*	Systematic Error
A1	346	0.000061	0.006064	0.51 %
A2	[0.510836, 0.510837]	0.000003	0.068650	0.00 %

**Table 9:** The estimates of *EER*, SEs, and 95 % CIs for high-accuracy Algorithm A1 and low-accuracy Algorithm A2

Algorithm	*EÊR*	*SÊ*	95 % Confidence interval
A1	0.006064	0.000301	(0.005511, 0.006703)
A2	0.068650	0.000743	(0.067174, 0.070162)

**Table 10:** The estimates of *TAR*s, SEs, and 95 % CIs for high-accuracy Algorithms B1 and B2, while *FAR* was specified at 0.001

Algorithm	*TÂR (f)*	*SÊ*	95 % Confidence interval
B1	0.993255	0.000325	(0.992622, 0.993922)
B2	0.989263	0.000470	(0.988307, 0.990159)

**Table 11:** The estimates of *EER*s, SEs, and 95 % CIs for relatively low-accuracy Algorithms B3 through B5

Algorithm	*EÊR*	*SÊ*	95 % Confidence interval
B3	0.012409	0.000378	(0.011638, 0.013148)
B4	0.012903	0.000360	(0.012205, 0.013609)
B5	0.013634	0.000338	(0.012940, 0.014287)

**Table 12:** The two-tailed *p*-values for Algorithms B1 and B2

Algorithm	*p*-value
B1	0.0000
B2	0.1049

**Table 13:** The average correlation coefficients of the statistic of interest *EER* out of ten runs among relatively low-accuracy Algorithms B3 through B5

Algorithm	B3	B4	B5
B3	1.000000	0.360888	0.398198
B4		1.000000	0.453439
B5			1.000000

**Table 14:** The two-tailed *p*-values of two statistics of interest *EER*s for Algorithms B3 through B5, where the correlation coefficient was taken into account

Algorithm	B3	B4	B5
B3	1.0000	0.2370	0.0019
B4		1.0000	0.0457
B5			1.0000

## Footnotes

1 Specific hardware and software products identified in this report were used in order to adequately support the development of technology to conduct the performance evaluations described in this document. In no case does such identification imply recommendation or endorsement by the National Institute of Standards and Technology, nor does it imply that the products and equipment identified are necessarily the best available for the purpose.
